# Epidemiology, management, and treatment outcomes of metastatic spinal melanoma

**DOI:** 10.1016/j.wnsx.2023.100156

**Published:** 2023-01-21

**Authors:** David X. Zheng, Sauson Soldozy, Kathleen M. Mulligan, Melissa A. Levoska, Erin F. Cohn, Ariel Finberg, Peter Alsaloum, Thomas B. Cwalina, Simon J. Hanft, Jeffrey F. Scott, Luke D. Rothermel, Vinod E. Nambudiri

**Affiliations:** a Department of Dermatology, University Hospitals Cleveland Medical Center, Case Western Reserve University, Cleveland, OH, United States; b Department of Surgery, Division of Surgical Oncology, University Hospitals Cleveland Medical Center, Case Western Reserve University, Cleveland, OH, United States; c Department of Neurological Surgery, University of Miami, Miami, FL, United States; d Department of Neurosurgery, Westchester Medical Center, New York Medical College, Valhalla, NY, United States; e Department of Genetics and Genome Sciences, Case Western Reserve University, Cleveland, OH, United States; f Department of Dermatology, Johns Hopkins University School of Medicine, Baltimore, MD, United States; g Department of Dermatology, Brigham and Women's Hospital, Harvard Medical School, Boston, MA, United States

**Keywords:** Melanoma, Spinal melanoma, Spinal metastasis, Neuroimmunology, Neurooncology, CAR, chimeric antigen receptor, CNS, central nervous system, HMB-45, human melanoma black 45, ICIs, immune checkpoint inhibitors, MEK, mitogen-activated protein kinase, MRI, magnetic resonance imaging, PD-1, programmed death receptor-1, SRS, stereotactic radiosurgery, T-VEC, talimogene laherparepvec

## Abstract

Metastatic spinal melanoma is a rare and aggressive disease process with poor prognosis. We review the literature on metastatic spinal melanoma, focusing on its epidemiology, management, and treatment outcomes. Demographics of metastatic spinal melanoma are similar to those for cutaneous melanoma, and cutaneous primary tumors tend to be most common. Decompressive surgical intervention and radiotherapy have traditionally been considered mainstays of treatment, and stereotactic radiosurgery has emerged as a promising approach in the operative management of metastatic spinal melanoma. While survival outcomes for metastatic spinal melanoma remain poor, they have improved in recent years with the advent of immune checkpoint inhibition, used in conjunction with surgery and radiotherapy. New treatment options remain under investigation, especially for patients with disease refractory to immunotherapy. We additionally explore several of these promising future directions. Nevertheless, further investigation of treatment outcomes, ideally incorporating high-quality prospective data from randomized controlled trials, is needed to identify optimal management of metastatic spinal melanoma.

## 1 Introduction

Melanoma metastases to the central nervous system (CNS) occur in 10–40% of melanoma patients depending on stage at diagnosis, rising up to 80% in autopsy series.^1^^,^^2^ In contrast to intracranial metastases, metastatic spinal melanoma is particularly rare, representing a late event in the evolution of the disease, with estimates for median overall survival of 4 months, 2-year survival of 0.2%, and 5-year survival of 0%.^3^, ^4^, ^5^ Surgery and radiotherapy are mainstays of treatment.^6^^,^^7^ While prognosis remains poor for patients with metastatic melanoma, the recent advent of immune checkpoint inhibitors (ICIs), including ipilimumab, nivolumab, and pembrolizumab, as well as targeted therapies, including the BRAF-inhibitors vemurafenib and dabrafenib, has heralded favorable increases in progression-free survival; however, the benefit of these therapeutic advances in the metastatic spinal melanoma population is unclear.^8^, ^9^, ^10^ Regardless, optimal treatment remains under debate, and may vary on a patient-by-patient basis depending on factors such as functional status, systemic disease burden, and time to spinal metastasis from primary tumor diagnosis.^1^^,^^3^^,^^6^^,^^11^ In light of the challenge presented by this disease process, we provide a review of the epidemiology, diagnosis, and management of metastatic spinal melanoma.

## 2 Methods and results

We performed a scoping review of PubMed, Scopus, and MEDLINE for articles published from inception to August 2021 using the search term “metastatic spinal melanoma.” Studies were included if they evaluated melanoma metastatic to the spine. Studies of primary spinal cord melanoma, review articles, and non-English manuscripts were excluded. References of included studies were reviewed to identify additional relevant articles. This study was exempt from institutional review board approval given the use of publicly available, deidentified data.

Overall, 359 non-duplicate articles were identified. Based on the above criteria, 213 articles were excluded after title/abstract and full-text review, resulting in 146 included articles. Among these included articles, there were 68 (46.6%) case reports, 61 (41.8%) retrospective or prospective cohort studies, and 17 (11.6%) basic science studies (Fig. 1). In the paragraphs to follow, we summarize these articles to review the current landscape of metastatic spinal melanoma, from its demographics to contemporary treatment methods.

**Figure:**
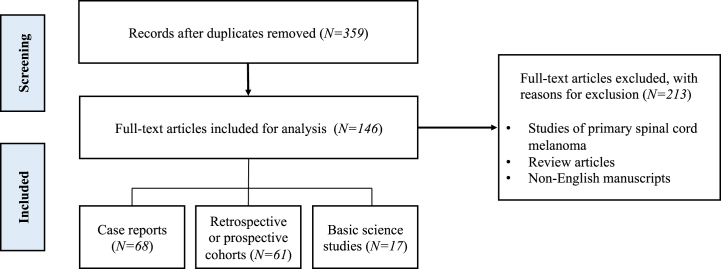
Flowchart of studies selected for inclusion and review.

### 2.1 Epidemiology

The demographics of metastatic spinal melanoma are similar to those of cutaneous melanoma, with a slight predominance in males (55–60%) and disease mainly occurring in White individuals (>95%) in worldwide population-based cohorts.^3^^,^^5^ Median age of primary tumor diagnosis is approximately 45 years, while median time to spinal metastasis is approximately 3 years.^3^^,^^5^ Metastases tend to present in more than one spinal region, typically favoring the thoracic and lumbar spines.^3^, ^4^, ^5^, ^6^ While cutaneous primary tumors tend to be most common (74–83%), followed by tumors of unknown primary site (14–19%),^3^^,^^4^ metastatic ocular and mucosal primary melanomas have also been reported.^7^^,^^12^, ^13^, ^14^ Over half of spinal metastasis patients also have visceral metastases, with lung metastases being most common.^5^^,^^6^ While clinical symptoms vary, pain (e.g., back, radicular) tends to be the most common (75–82%) presenting feature, and neurologic deficits (e.g., weakness, paresthesia, incontinence) are seen in 17–21% of patients.^4^^,^^5^^,^^15^ One cohort study comprising patients from ten different countries reported a higher incidence of metastatic spinal melanoma in the West compared to in Asian countries.^16^

### 2.2 Diagnostic workup

While magnetic resonance imaging (MRI) appears to be the most commonly employed initial diagnostic test, bone scintigraphy (i.e., radionuclide bone scanning) is another suitable option for identifying spinal metastasis given the ease of performing a whole-body survey and its high sensitivity (62–100%) and specificity (78–100%) for spinal metastasis.^17^, ^18^, ^19^, ^20^ However, bone scintigraphy may miss osteolytic metastatic lesions, as are most often seen in metastatic spinal melanoma.^20^ MRI also appears to be more sensitive and specific for spinal metastases than bone scintigraphy in certain regions of the spine, including the lumbar region, which tends to be favored by metastatic melanoma.^3^, ^4^, ^5^^,^^19^ On MRI, the classic finding in metastatic spinal melanoma is a small (<1.5 cm) lesion with accompanying vasogenic edema, typically hyperintense on T1 sequences secondary to hemorrhage and hypointense on T2/FLAIR, with post-contrast T1 imaging demonstrating either peripheral rim enhancement or diffuse heterogenous enhancement.^13^^,^^21^

Regardless, definitive diagnosis of metastatic spinal melanoma is usually made with needle biopsy, or after post-surgical pathological studies or autopsies.^13^^,^^23^^,^^24^ Immunopositivity for S100, vimentin, and/or human melanoma black 45 (HMB-45) have proven useful in supporting cytological diagnosis.^15^^,^^22^ Li et al found vimentin to be significantly upregulated in a mouse model of melanoma metastatic to the lung, also implicating its potential as a biomarker for hematogenous spread.^25^ HMB-45 is an anti-melanoma antibody signifying active melanosome formation, and therefore helps to confirm diagnosis of melanoma.^26^ Gokaslan et al additionally demonstrated the utility of matrix metalloproteinases 2 and 9, as well as urokinase-type plasminogen activator, as tumor markers with higher levels of activity in metastatic spinal melanoma compared to primary spinal melanoma.^27^^,^^28^

### 2.3 Surgery and radiotherapy

Treatment for metastatic spinal melanoma is similar to that for other metastatic tumors of the spine, incorporating steroids, spinal precautions, decompressive surgical intervention with or without spinal fixation, and radiotherapy.^6^^,^^7^ Surgical resection improves patient quality-of-life by reducing sequelae due to mass effect (e.g., neurological symptoms).^29^ One randomized trial of decompressive surgery plus postoperative radiotherapy versus radiotherapy alone in patients with metastatic spinal cord tumors showed that the surgery group was able to walk longer (median 122 days; 13 days in patients receiving radiotherapy alone), and improved motor function and continence.^30^ While metastatic spinal melanoma tends to present as smaller (<1.5 cm) lesions,^13^^,^^21^ surgical resection also facilitates removal of tumors ≥2 cm.^29^^,^^31^

However, in part due to small sample sizes reported in the literature, the effect of surgery on survival in metastatic spinal melanoma is poorly established.^4^^,^^6^^,^^13^^,^^32^ In a retrospective analysis of 64 patients with metastatic spinal melanoma receiving decompressive surgical intervention, the largest such cohort to date, Sellin et al found median overall survival of 5.7 months.^6^ They also showed that systemic disease at time of surgery (median overall survival 1.6 months; 11.2 months in patients without systemic disease) and total spinal disease burden ≥3 vertebral levels (median overall survival 3.3 months; 9.4 months in patients with disease burden <3 vertebral levels) were significantly associated with worse overall survival.^6^ Notably, the median overall survival of 5.7 months reported by Sellin et al exceeds that of 3.6 months reported by Gokaslan et al in their analysis of 133 metastatic spinal melanoma cases from the same institution more than two decades earlier.^4^^,^^6^ This suggests that survival outcomes have improved over time at least at one institution, potentially also in part due to development of targeted medical therapies, advances in radiotherapy, and earlier diagnosis.

Hadden et al reviewed outcomes of 39 metastatic spinal melanoma patients with cord compression and additionally demonstrated that perioperative lactate dehydrogenase ≤8 μkat/L, preoperative hemoglobin >11.5 mg/dL, ≥4 years between melanoma diagnosis and presentation of spinal metastasis, and Eastern Cooperative Oncology Group Performance status ≤2 were all associated with increased survival.^11^ While metastatic spinal melanoma presents in relatively young patients, who likely are reasonable surgical candidates, prognosis remains quite poor and spinal metastasis is typically a manifestation of widely disseminated disease from a biologically aggressive tumor.^6^^,^^15^ Thus, neurosurgeons should remain mindful of the aforementioned factors predicting worse overall survival, as well as critical in their recommendation of aggressive surgical intervention.

### 2.4 Stereotactic radiosurgery

As discussed, radiotherapy has also been established as a mainstay of metastatic spinal melanoma treatment in addition to decompressive surgical intervention.^33^, ^34^, ^35^ However, melanoma is traditionally considered to be a radioresistant malignancy, especially to standard external beam fractionated dosing regimens.^36^ Even despite conventional external beam radiotherapy, metastatic spinal melanoma tends to progress due to both lack of precision in delivering large single-fraction doses and the spinal cord's low radiation tolerance, which limits treatment doses to levels far below the optimal therapeutic dose.^37^ Moreover, the vascularity of metastatic spinal melanoma complicates surgical excision.^37^^,^^38^ Stereotactic radiosurgery (SRS), which involves the delivery of high doses of single-fraction radiation to a specific target, has emerged as a promising approach in operative treatment of metastatic spinal melanoma, addressing several of these limitations.^39^ Its benefit lies in the ability to target both individual tumor cells and tumor vasculature, resulting in an intense anti-tumor immune response via release of proinflammatory cytokines.^40^ SRS also facilitates the precise delivery of target volumes of radiation, therefore optimizing the preservation of healthy tissue nearby.^41^ Few studies exist detailing metastatic spinal melanoma outcomes following SRS, but the limited data are promising. Caruso et al summarize these data,^39^ finding that SRS provides high rates of both local tumor control (75–100%) and pain relief (96–100%) in patients with metastatic spinal melanoma.^42^, ^43^, ^44^, ^45^ These benefits, however, must be weighed against complications from SRS such as radiation-induced myelopathy, vertebral compression fractures, and radiculopathy.^39^

### 2.5 Contemporary systemic therapies

The development of contemporary systemic therapies, including of ICI and targeted therapy within the last decade, has revolutionized treatment of metastatic melanoma. Among these are ICIs targeting programmed death receptor-1 (PD-1) and its ligand (e.g., nivolumab, pembrolizumab) or cytotoxic T-lymphocyte antigen-4 (e.g., ipilimumab), as well as targeted therapies against melanoma with the BRAF V600E mutation that confers increased risk of CNS metastasis (e.g., vemurafenib, dabrafenib).^2^^,^^39^ Immunotherapy portends improved progression-free survival in patients with melanoma metastatic to the CNS,^8^^,^^9^ but prognosis remains poor, and the optimal management of patients with metastatic spinal melanoma likely entails individualized treatment combining surgery, radiotherapy, and immunotherapy.^39^^,^^46^

Nivolumab and pembrolizumab are approved as first-line treatments for wild-type BRAF melanoma, and first- or second-line treatments for BRAF-mutated melanoma, but phase III trials demonstrating their efficacy do not present data with enough granularity such that outcomes for metastatic spinal melanoma in particular may be discerned.^47^^,^^48^ Similarly, combination of BRAF inhibitors with mitogen-activated protein kinase (MEK) inhibitors (e.g., binimetinib) has resulted in longer progression-free and overall survival (up to 3 years in patients with advanced disease) and less toxicity compared to BRAF inhibitors alone, yet outcomes specifically for metastatic spinal melanoma have yet to be elucidated.^49^, ^50^, ^51^ Triple therapy involving first-line anti-PD-1 antibodies plus BRAF/MEK inhibitors has been investigated, but this is not routinely used clinically due to higher toxicities and lack of evidence demonstrating improved outcomes for triple therapy over the current standard of sequential therapy.^52^ New targets continue to be identified, contributing to the ever-shifting landscape of ICIs for metastatic melanoma. In a recent phase II-III trial comparing relatlimab (lymphocyte activation gene-3 antibody) plus nivolumab versus nivolumab alone, the combination therapy provided a greater benefit in progression-free survival than nivolumab alone, with minimal toxicity.^53^

Given the relatively recent advent of immunotherapy for metastatic melanoma, and the rarity of metastatic spinal melanoma in particular, data on systemic therapy treatment outcomes remain scarce. In a phase I study of high-dose interleukin-2 and SRS, Seung et al found that five of seven (71.4%) patients with metastatic spinal melanoma had a partial or complete response to therapy.^39^^,^^54^ In a retrospective analysis of 37 patients with metastatic spinal cord compression (21 non-small cell lung cancer, 9 renal cell carcinoma, 7 melanoma) receiving both PD-1 inhibition and radiotherapy, all patients reported pain relief and 46% of patients reporting numbness/motor symptoms at baseline reported improvement.^55^ While outcomes are not stratified by tumor type, precluding analysis of metastatic spinal melanoma in particular, these data suggest that palliative radiotherapy is well tolerated and effective in patients with metastatic cord compression treated with anti-PD-1 therapy. High-quality prospective data on treatment outcomes for immunotherapy in metastatic spinal melanoma are needed, although this need is complicated by its generally low incidence and the fact that patients with melanoma metastatic to the CNS are often excluded from clinical trials (e.g., concerns of poor overall survival, emergent need to initiate radiation for patients with symptomatic spinal metastasis).^2^^,^^55^

While the effect of specific immunotherapies on metastatic spinal melanoma outcomes requires further investigation, Shankar et al performed a 3-year retrospective analysis of surgical outcomes for 18 metastatic spinal melanoma patients treated at their institution, of whom 8 had a history of immunotherapy exposure and 10 were immunotherapy naïve.^56^ Interestingly, patients with a history of immunotherapy exposure had significantly shorter overall survival (median 98 days; 315 days in immunotherapy naïve patients). The authors concluded that immunotherapy failure resulting in progressive spinal disease may represent a particularly aggressive time point in the natural history of melanoma progression, and they hypothesize that immunological selective pressure resulting from immune checkpoint inhibition may facilitate “tumor escape” via immunoediting, leading to aggressive recurrence.^56^^,^^57^ We additionally encourage researchers to further assess the effect of immunotherapy failure on outcomes in metastatic spinal melanoma.

### 2.6 Future directions

Despite advances in management, survival outcomes for metastatic spinal melanoma remain poor. However, new therapeutics are being developed to inhibit metastatic progression by targeting steps of the metastatic cascade including tumor cell intravasation into vasculature, extravasation into secondary tissue, and colonization of metastatic sites. For example, disrupting endothelial-tumor cell interactions mediated by the ephrin-B2-EphB4 pathway protects against hematogenous spread of melanoma cells to the spine in a mouse model of spinal metastasis.^58^ Additional work in mice has shown that increasing the expression of nuclear transport factor 2 in metastatic melanoma cells reduces cell motility and metastasis.^59^ Kratzsch et al demonstrate that targeting tumor growth and tumor cell interactions with vasculature using inhibitors of mammalian target of rapamycin and vascular endothelial growth factor receptors may delay development of symptomatic spinal metastasis, though therapeutic efficacy was only mild.^60^ Collectively, these studies suggest that elucidating the molecular underpinnings of the metastatic cascade could reveal novel approaches for prophylactic treatment of metastatic spinal melanoma.

Additionally, new systemic therapies for treatment of metastatic melanoma continue to emerge. Oncolytic virotherapy, which involves intratumoral delivery of genetically engineered oncolytic virus (e.g., talimogene laherparepvec [T-VEC], a modified herpes simplex virus), has shown promise in treating melanoma metastatic to the CNS via an abscopal effect.^2^ While T-VEC is the most well-studied of these treatments, other oncolytic viral vectors are currently being investigated for their potential in treating metastatic CNS melanoma.^61^ Other approaches include tumor-infiltrating lymphocyte therapy, which utilizes expansion of a patient's autologous T-cells, manipulation *ex vivo*, and re-infusion into the patient to generate an anti-tumor response. This method has shown durable, complete response rates in up to 40% of patients with melanoma metastatic to the CNS who failed other immunotherapies.^62^ Finally, chimeric antigen receptor (CAR) T-cell therapy has shown promise in the treatment of brain metastases and primary brain tumors, and may therefore demonstrate efficacy specifically in treating melanoma metastatic to the CNS.^63^^,^^64^ CAR T-cell therapy involves genetically altering patient T-cells with viral vectors to express CAR constructs. Newly engineered T-cells are then expanded *ex vivo* and re-injected into the patient, where they exert anti-tumor effects.^65^

Despite the clinical success of ICIs in treating metastatic melanoma, challenges remain for tumors refractory to immunotherapy.^52^ Mechanisms conferring resistance to anti-PD-1 therapy are currently being investigated, and targets for combination therapy have been identified. One such target is sphingosine kinase-1, which is overexpressed in many tumors including melanoma, and has been shown to reduce efficacy of anti-PD-1 therapy by increasing expression of factors involved in immunosuppression.^66^ In addition to mechanisms of resistance originating from tumors themselves, external factors such as the gut microbiome have been shown to influence the tumor microenvironment and regulate response to anti-PD-1 therapy. Modulating these external factors, including the composition of the gut microbiome itself, may therefore represent an alternative treatment approach for tumors refractory to anti-PD-1 therapy. Importantly, Baruch et al and Davar et al observed evidence of clinical improvement in patients with PD-1-refractory melanoma who received fecal microbiota transplantation from patients successfully responding to anti-PD-1 therapy.^67^^,^^68^ As mechanisms for anti-PD-1 therapy resistance continue to be clarified, combination regimens will likely need to be tailored to subsets of treatment-refractory patients.

## 3 Conclusions

Understanding of the current melanoma literature is imperative when discussing treatment options with metastatic spinal melanoma patients. While surgery achieves decompression and/or stabilization with symptom improvement, leading to improved quality of life, its impact on survival is not clearly established. A multifaceted treatment approach tailored to each patient, their unique symptom presentation, and overall disease burden should be considered. While outcomes have improved over recent years, particularly with the advent of immunotherapies for use in conjunction with surgery and radiotherapy, metastatic spinal melanoma remains an aggressive disease process with poor prognosis. For this reason, new treatment options continue to be studied, especially for patients with disease refractory to immunotherapy. Further research on outcomes, ideally including prospective data from clinical trials, is needed to identify and further refine management strategies for patients with metastatic spinal melanoma.

## Funding declaration

The authors report no funding sources relevant to this work.

## Data availability statement

Data sharing not applicable to this article as no datasets were generated or analyzed during the current study.

## Declaration of competing interest

The authors declare that they have no known competing financial interests or personal relationships that could have appeared to influence the work reported in this paper.
